# Trial of labour versus elective caesarean delivery for estimated large for gestational age foetuses after prior caesarean delivery: a multicenter retrospective study

**DOI:** 10.1186/s12884-023-05688-1

**Published:** 2023-05-26

**Authors:** Matthieu Chamagne, Maêva Bôle Richard, Alexandre Vallee, Jellila Tahiri, Bruno Renevier, Sandra Dahlhoff, Diane Garcia, Alexandre Vivanti, Jean Marc Ayoubi

**Affiliations:** 1 Department of Obstetrics and Gynecology, Foch Hospital, 92150 Suresnes, France; 2 Department of Clinical Research and Innovation, Foch Hospital, 92150 Suresnes, France; 3 Department of Obstetrics and Gynecology Centre Hospitalier Universitaire, Hôpital Archet II, Pôle “Femme-Mère-Enfant’’, Nice, France; 4 Department of Obstetrics and Gynecology, André Grégoire Hospital, 93100 Montreuil, France; 5 Department of Obstetrics and Gynecology, Mercy Hospital, 57530 Ars-Laquenexy, France; 6 Division of Obstetrics and Gynecology, DMU Santé Des Femmes Et Des Nouveau-Nés, Antoine Béclère Hospital, Paris Saclay University, AP-HP, 92140 Clamart, France

**Keywords:** Trial of labour after caesarean delivery, Estimated large for gestational age, Macrosomia, Scarred uterus

## Abstract

**Background:**

Lower rates of successful trial of labor after cesarean (TOLAC) in association with fetal macrosomia were previously reported. This study aimed to compare TOLAC to elective caesarean delivery (CD) in women with estimated fetal weight large for gestational age (eLGA) and a prior CD. Primary outcome was to analyse the mode of delivery in case of TOLAC. Secondary outcome was to compare maternal and foetal morbidity.

**Methods:**

We conducted a retrospective, descriptive, multicentric, cohort study in five maternity units between January and December 2020. Inclusion criteria were: women with a single prior CD and eLGA or neonatal weight > 90th percentile with singleton pregnancy and gestational age ≥ 37 weeks. Main outcome measures: rate of vaginal delivery, maternal and fetal morbidity including: shoulder dystocia, neonatal hospitalization, fetal trauma, neonatal acidosis, uterine rupture, 3^rd^ and 4^th^ perineal tears, post-partum hemorrhage, and a need for blood transfusion.

**Results:**

Four hundred forty women met inclusion criteria, including 235 (53.4%) eLGA. 170 (72.3%) had a TOLAC (study group) and 65 (27.7%) an elective CD (control). 117 (68.82%) TOLAC had a vaginal delivery. No significant differences were found between the two groups in the rates of: postpartum haemorrhage, transfusion, Apgar score, neonatal hospitalization, and foetal trauma. Cord lactate was higher in the case of TOLAC (3.2 vs 2.2, *p* < 0.001). Median fetal weight was 3815 g (3597–4085) vs. 3865 g (3659–4168): *p* = 0.068 in the study vs. controls group respectively.

**Conclusion:**

TOLAC for eLGA fetuses is legitimate because there is no difference in maternal–fetal morbidity, and the CD rate is acceptable.

## Background

Macrosomia is classically defined as a birth weight greater than 4000 g at term. Currently, a foetus is considered macrosomic when its weight is greater than the 90th percentile for gestational age according to reference curves for a given population [1–4]. The incidence of macrosomia ranges from 5 to 10% [1–4]. In France, according to the 2021 National Perinatal Survey, the rate of suspected foetal macrosomia is estimated to be 8,7% [5]. Obstetricians are increasingly dealing with the delivery of suspected macrosomic foetuses. Weiner et al. noted that the caesarean delivery (CD) rate is doubled when macrosomia is suspected before delivery [6].

The 2021 French National Perinatal Survey found a proportion of multiparous women with a history of CD of 20.7%. Trial of labour after caesarean deliveries (TOLAC) is attempted in 68.9% of patients, 73.3% of whom actually give birth vaginally. Previous studies have found that the risk of CD in case of macrosomia was increased [7–9] but there are few recent publications [10, 11]. The CD rate has been stable since 2010 in France. It was evaluated at 21.4% according to the 2021 Perinatal Survey. In 2015, the World Health Organization (WHO) recommended a percentage of CD between 10 and 15% based on the fact that CD was effective in reducing maternal and foetal mortality but only when medically justified. CD rates above 10% were not associated with a reduction in maternal and neonatal mortality rates.

The latest French recommendations for the scarred uterus were established by the Collège National des Gynécologues Obstétriciens Français (CNGOF) in 2012. They report an increased failure rate of vaginal delivery attempt and a doubled risk of uterine rupture in case of macrosomia. However, due to an estimated success rate of over 60% in this situation, the recommendations allow a vaginal delivery attempt for an estimated foetal weight up to 4500 g.

The question the obstetrician faces is whether to accept a TOLAC or to schedule a CD in case of eLGA and a scarred uterus. Literature regarding the preferred mode of delivery in this situation is scarce.

Because of the independent increase in the risk of CD in cases of macrosomia and a scarred uterus, it appears interesting to analyse the mode of delivery of patients with a scarred uterus and estimated foetal weight large for gestational age (eLGA) as well as the associated maternal-foetal morbidity [7, 12]. This study aimed to compare maternal and neonatal morbidity of TOLAC to an elective CD in women with eLGA and a prior caesarean delivery. The secondary purpose was to identify risk factors for failed TOLAC in case of eLGA.

## Material and methods

A retrospective, descriptive, multicentric, cohort study was conducted in five maternity units (three tertiary care centers and two level II centers) between January and December 2020. All women with a scarred uterus and eLGA or neonatal weight > 90^th^ percentile were included. Inclusion criteria were singleton pregnancy, and gestational age > 37 weeks for delivery. Women with a history of multi-scar uterus, non-cephalic presentation, multiple pregnancy, intrauterine foetal demise, or maternal contra indication for TOLAC, uterine scars other than caesarean section (uterine surgery), and corporeal incisions were excluded. 

eLGA was suspected by sonography which was performed between 36 and 41 weeks. Estimation of foetal weight was calculated using the Hadlock equation (head circumference, femur length and abdominal circumference). Suspected large estimation of foetal weight was defined as a foetal weight above the 90^th^ percentile using the « Collège Français d’Echographie Fœtale» (CFEF) curves [13].

TOLAC was accepted in case of estimated foetal weight < 4500 g in accordance with the French recommendation of the CNGOF. Failure to progress was decided after 3 h at the same dilatation (with active management defined as uterine contractions and membrane rupture) in the active phase of labour. In the case of labour induction, cervical ripening was achieved by a Foley catheter or a Cook balloon.

Indication of elective CD was rejection of TOLAC by the patient or an unfavorable cervix with a medical decision not to attempt a TOLAC.

The primary outcome was the mode of delivery in case of TOLAC. Secondary outcomes were maternal and foetal morbidity including shoulder dystocia, neonatal hospitalization, foetal trauma, neonatal acidosis, uterine rupture, 3^rd^ and 4^th^ perineal tears, post-partum haemorrhage, and a need for blood transfusion. This was not a composite criterion. These data were obtained from the patients' pregnancy and delivery records.

### Statistical analysis

Continuous variables are presented as median + interquartile range (IQR) and were compared using an independent T-test or Mann–Whitney test. Categorical variables are presented as n (%) and were compared using a Chi-squared test or Fisher’s exact test. Variables associated with *p* values lower than 0.20 were included in a multiple stepwise regression with forward—backward method. The ability of the multiple logistic regression models to allow discrimination was quantified by the area under the ROC curve (AUC). *P* value < 0.05 was considered as significant. All analyses were performed using SAS software (version9.4; SAS Institute, Carry, NC).

The study protocol was approved by the « Foch hospital ethical committee», Suresnes, France (1/4/2021). Protocol number: IRB00012437. Because of the retrospective nature of the study, a non-opposed consent was obtained from patients. All methods were performed in accordance with the relevant guidelines and regulations.

## Results

**Fig. 1:**
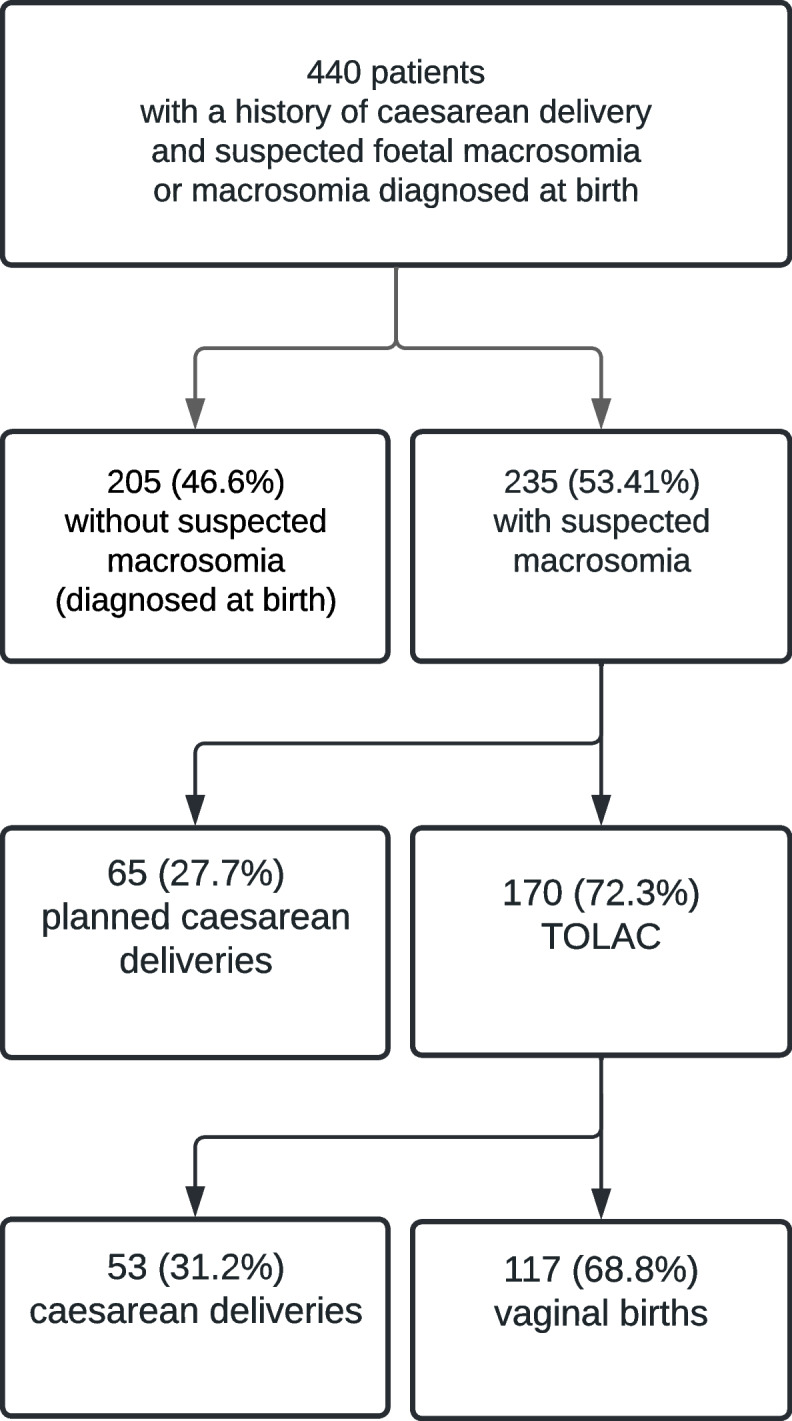
Flow chart

There were 17,675 births total in the five centers during the year 2020. 440 women met the inclusion criteria; the global caesarean section rate was 45.2% [40.6 – 49.9] (Fig. 1: Flow chart). During the study period, the rate of CD in the five centers were respectively 19; 20.7, 22, 22 and 25.8% (all births included).

Two hundred and thirty five patients (53.4%) had eLGA: 170 (72.3%) had a TOLAC (study group) and 65 (27.7%) an elective CD (control). The main indication of the prior CD in the study group were cardio-tocographic (CTG) abnormality (68.82%) and stagnation (16.47%). In the control group, the main indications were stagnation (75.38%) and CTG abnormality (21.54%). The women’s characteristics are summarized in Table 1.

**Table 1:** Patients characteristics

	**Elective C-section (*n* = 65)**	**TOLAC (*n* = 170)**	***P* value**
**Age (years)**	34(31–38)	33(30–36.25)	0.051
**Size (height)**	1.54(1.58–1.675)	1.65(1.6–1.69)	0.168
**BMI (kg/m**^**2**^**)**	28.4(23.9–33.6)	26.5(23.7–30.4)	0.078
**Weight gain during pregnancy (kilos)**	14(7–18)	13(9–16)	0.769
**Pre-existing diabetes**	3(4.69%)	3(1.78%)	0.210
**Gestational diabetes**	29(46.03%)	76(45.51%)	0.943
**Prior vaginal delivery**	9(13.85%)	62(36.47%)	< 0.001
**Estimated foetal weight > 90**^**th**^** percentile**^**a**^	65(100%)	170(100%)	
**Estimated foetal weight > 4000 g**	13(20%)	14(8.24%)	0.011

*BMI* Body Mass Index

^a^According to the Collège Français d’Echographie fœtale curves

One hundred and seventeen (68.82%) women with a TOLAC and eLGA had a vaginal delivery (vaginal birth after caesarean: VBAC). No significant differences between the two groups were found in the rate of uterine rupture, postpartum haemorrhage, transfusion, Apgar score, neonatal hospitalization, and foetal trauma. Cord lactate was higher in the case of TOLAC (3.2 vs 2.2, *p* < 0.001). Estimated foetal weight above 4000 g was more frequent in the elective C-section group (*p* = 0.011) but there was no significant difference concerning birthweight between the two groups (*p* = 0.068) (Table 2).

**Table 2:** Comparison of Maternal and Perinatal Outcome between elective caesarean section and TOLAC in case of eLGA

	**Elective C-section (*n* = 65)**	**TOLAC (*n* = 170)**	***P* value**
**Birth weight**	3865 (3659–4168)	3815 (3597–4085)	0.068
**Birth weight > 90**^**th**^** percentile**	61 (93.85%)	146 (85.88%)	0.074
**Gestational age (weeks)**	38.92	39.42	< 0.001
**pH**	7.27 (7.23–7.30)	7.26 (7.22–7.31)	0.763
**Lactates**	2.2 (1.7–3.2)	3.2 (2.4–4.6)	< 0.001
**5-min Apgar score**	10 (10–10)	10 (10–10)	0.04
**Shoulder dystocia (needing Jacquemier)**	0	4 (2.42%)	
**Hospitalization in neonatology**	4 (6.15%)	7 (4.12%)	0.519
**Intubation**	1 (1.54%)	1 (0.59%)	0.503
**VNI**	8 (12.31%)	10 (5.88%)	0.112
**Fracture (clavicle)**	1 (1.54%)	3 (1.76%)	0.904
**Brachial plexus**	0	1	0.419
**Foetal death**	0	0	
**Intra-cerebral haemorrhage**	0	0	
**Hypoglycemia**	2 (3.08%)	4 (2.35%)	0.757
**Phototherapy**	2 (3.08%)	5 (2.96%)	0.962
**VBAC**	0	117 (68.8%)	
**Uterine rupture**	0	0	
**Postpartum haemorrhage**	10 (15.38%)	15 (8.82%)	0,158
**Blood transfusion**	1 (1.54%)	1 (0.59%)	0.503
**Episiotomy**	0	9 (5.49%)	0.025
**3**^**RD**^** / 4**^**TH**^** perineal tear**	0	0	
**Operative delivery**	4 (6.15%)	34 (20%)	0.005
**CD complications**	4 (6.45%)	6 (5.13%)	0.542

*VNI* non-invasive ventilation, *VBAC* vaginal birth after caesarean delivery, *CD* caesarean delivery

**Fig. 2:**
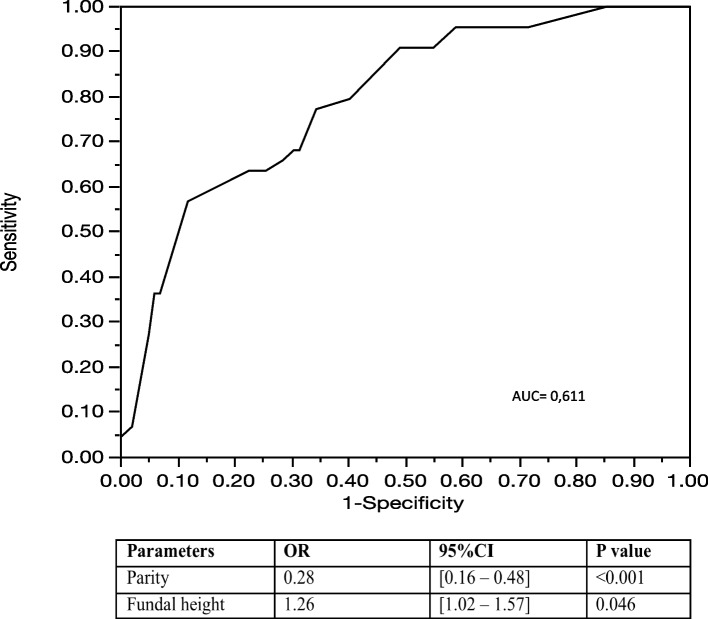
Receiver operative characteristics (ROC) curve for predicting vaginal birth after caesarean section in case of eLGA. AUC = 0.611

Patients who had a successful VBAC were significantly taller (1.65 vs 1.63 m *p* = 0.01), had a smaller fundal height (33 vs 34 cm *p* = 0.04) and had had a prior vaginal delivery (*p* < 0.001). The main indication for CD was a failure of progress (75.3%). In multivariate analysis including prior vaginal delivery, fundal height, parity and gestational age, the predictive factors for failed TOLAC were fundal height > 34 cm and no prior vaginal delivery (Fig. 2: Receiver operative characteristics (ROC) curve for predicting vaginal birth after caesarean section in case of eLGA. AUC = 0.611)*.*

The rate of operative vaginal delivery was 20% with a failure rate of 8.8% (indicating a CD).

There was no significant difference in the rate of successful delivery whether or not macrosomia was suspected (68.8 vs 68.2% *p* = 0.9).

## Discussion

This study showed that 69% of women with eLGA who underwent a TOLAC had a vaginal delivery. Such figures remain consistent with known data about trial of labour after a caesarean section (CS) [14, 15]. According to a 2023 nationwide register-based cohort study in Finland, the rate for VBAC was 67% [16]. But it varies from country to country: 39 to 70% in the United States [17].

With respect to the complications in the case of TOLAC versus an elective CS, no differences were highlighted in case of eLGA. The usual complications of TOLAC, which are notably uterine rupture, hysterectomy, low APGAR score, foetal trauma, and neonatal hospitalization [18–23], were not more frequent in case of eLGA in a recent retrospective study except for the rate of post-partum haemorrhage [24].

Thus, there were no hysterectomies or transfusions among the patients in this study, compared with a rate of hysterectomy of 0.2%, and a rate of transfusion of 1.7% in the Rossi meta-analysis [25].

However, three of the 352 patients who underwent a TOLAC (0.85%) had a uterine rupture while the rate was 0.6% in the Chauhan meta-analysis with 142,075 patients with undefined estimated foetal weight [26]. The three cases of uterine rupture concerned unsuspected macrosomic infants. This could mean that the obstetrical team was more careful in case of eLGA.

In our study, there was no significant difference regarding the mode of delivery between actual macrosomia and eLGA in case of ‘real’ LGA. Unlike Weiner et al. [5], eLGA does not appear as a risk factor for CD in our study.

Furthermore, as identified in the Vikhareva study, the main indication for CD after TOLAC was the failure of progression of labor [27].

The odds of TOLAC were affected by previous vaginal delivery, maternal height, and fundal height in the univariate analysis. Numerous studies confirm the notion of previous vaginal delivery as a positive predictive factor as shown in the Wu meta-analysis [28]. The fundal height is analysed as a negative predictive factor in other studies such as the Iglesias-Benavides study [29]. In contrast, maternal height is not a frequently reported predictive factor: no difference in height is noted in the Levin study between successful and failed TOLAC [30], while Grobman’s results are consistent with this study [31].

## Conclusion

In case of eLGA, TOLAC is a safe option since there is no difference in maternal-foetal morbidity. The CD rate is reasonable.

## Data Availability

All data generated or analysed during this study are included in this published article.

## Abbreviations

CD: Caesarean delivery
TOLAC: Trial of labour after caesarean delivery
WHO: World health organization
eLGA: Estimated foetal weight large for gestational age
CFEF: Collège Français d’échographie foetale
CNGOF: Collège national des gynécologues obstétriciens français
IQR: Interquartile range
ROC: Receiver operative characteristics
AUC: Area under curve
